# Differences in Tri‐Trophic Community Responses to Temperature‐Dependent Vital Rates, Thermal Niche Mismatches and Temperature‐Size Rule

**DOI:** 10.1111/ele.70022

**Published:** 2024-12-02

**Authors:** Samuel Dijoux, Aslak Smalås, Raul Primicerio, David S. Boukal

**Affiliations:** ^1^ Department of Ecosystems Biology, Faculty of Science University of South Bohemia České Budějovice Czech Republic; ^2^ Czech Academy of Sciences, Biology Centre Institute of Entomology České Budějovice Czech Republic; ^3^ Department of Arctic and Marine Biology, Faculty of Biosciences, Fisheries and Economy, UiT The Arctic University of Norway Tromsø Norway; ^4^ SNA‐Skandinavisk naturoveråking AS (Scandinavian Nature‐Monitoring), DNV Tromsø Norway

**Keywords:** alternative stable states, emergent Allee effect, metabolic ecology, temperature‐size rule, thermal niche mismatch, trophic chain, warming

## Abstract

Warming climate impacts aquatic ectotherms by changes in individual vital rates and declines in body size, a phenomenon known as the temperature‐size rule (TSR), and indirectly through altered species interactions and environmental feedbacks. The relative importance of these effects in shaping community responses to environmental change is incompletely understood. We employ a tri‐trophic food chain model with size‐ and temperature‐dependent vital rates and species interaction strengths to explore the role of direct kinetic effects of temperature and TSR on community structure along resource productivity and temperature gradients. We find that community structure, including the propensity for sudden collapse along resource productivity and temperature gradients, is primarily driven by the direct kinetic effects of temperature on vital rates and thermal mismatches between the consumer and predator species, overshadowing the TSR‐mediated effects. Overall, our study enhances the understanding of the complex interplay between temperature, species traits and community dynamics in aquatic ecosystems.

## Graphical abstract

Our study investigates how tri‐trophic community respond differently to the direct effects of warming and resource productivity. Our model covers the main processes driving the dynamics and structure of tri‐trophic chains under the direct influences of warming, i.e. through the temperature‐dependent vital rates of species mediated by the temperature performance curves (TPC) and through the temperature‐induced reduction in body size, also known as the temperature‐size rule (TSR). Using multiple scenarios, we summarise different responses of size‐structured trophic chains across environmental gradients by considering the joint and separate influences of TPC and TSR in consumer and predator traits, when their thermal niches partially or fully overlap.

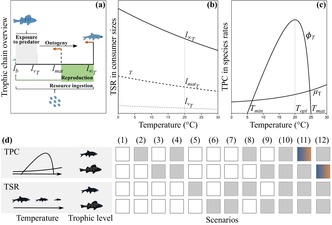

## 1 Introduction

Aquatic communities face numerous anthropogenic stressors including climate warming, habitat degradation, overharvesting, agricultural runoffs and invasions (IPBES 2019; Sala et al. 2000). Climate projections indicate a rise in global temperatures of +1.4°C–4.4°C by the year 2100 (IPCC 2023), which will profoundly impact community composition and structure worldwide (Sala et al. 2000; Young et al. 2016), particularly in aquatic ecosystems due to the high prevalence of ectotherms (Forster, Hirst, and Atkinson 2012).

Communities respond to warming through direct and indirect effects of temperature on individual traits, population dynamics and species interactions (Boukal et al. 2019; Lindmark et al. 2019; Uszko et al. 2017). The direct effects on vital rates (i.e., growth, consumption, metabolism and reproduction), characterised by nonlinear thermal performance curves (hereafter TPCs; Huey and Kingsolver 1989), delimit thermal niches and population dynamics of individual species (Angilletta 2009) and indirectly affect the dynamics of other interacting species. Thermal niche mismatches, characterised by incomplete thermal niche overlap between interacting species, can alter predator–prey dynamics (Álvarez‐Codesal et al. 2023; Dee et al. 2020) and likely have profound consequences on community structure beyond the well‐known phenological mismatches (Pörtner and Farrell 2008; Bideault et al. 2020), but their role in more complex communities has not been studied.

Warming also affects size thresholds that underlie life histories (Ohlberger 2013), such as maturation size (Niu et al. 2023) or maximum body size (Bazin et al. 2023). The pattern of declining body size with warming in many aquatic ectotherms is known as the ‘temperature‐size rule’ (hereafter TSR) (Daufresne, Lengfellner, and Sommer 2009) and is recognised as the ‘third universal response to warming’ (Forster, Hirst, and Atkinson 2012; Gardner et al. 2011). Warming‐induced changes in body size can indirectly affect predator–prey size ratios and alter species interactions (Boukal et al. 2019; Sentis et al. 2024). Size‐structured interactions can also cause abrupt shifts in community structure along environmental gradients (Lindmark et al. 2019; Thunell et al. 2021) due to emergent Allee effects associated with abrupt changes in population size structure (de Roos and Persson 2002), and these shifts can be triggered by changing body size (Dijoux and Boukal 2021).

The consequences of these combined effects of warming on species interactions and community structure remain incompletely understood as experiments aimed at separating the kinetic effects of temperature and the effects of TSR in size‐structured communities are challenging (Sentis et al. 2024; but see Bazin et al. 2024). Multiple recent models consider the effects of warming on species rates alone (Binzer et al. 2016; Dijoux et al. 2024; Fussmann et al. 2014; Uszko et al. 2017), together with TSR (Osmond et al. 2017; Sentis, Binzer, and Boukal 2017) or with thermal niche mismatches (Dee et al. 2020). However, they neglect population structure and ontogenetic variation in individual responses to temperature, which can determine population and community responses to warming (Gårdmark and Huss 2020). Other studies based on multi‐species size spectra models (Lindmark et al. 2022; Reum et al. 2024), multi‐species age‐structured models (Audzijonyte et al. 2013) and stage‐structured models (Lindmark et al. 2019; Thunell et al. 2021) show that warming can have qualitatively different consequences when it affects processes related to energy acquisition (e.g., intake rates) as opposed to energy expenditure and biomass loss (e.g., metabolic rate, mortality rate) (Lindmark et al. 2022; Reum et al. 2024). However, these studies mostly focus on specific warming scenarios and hence assume a monotonic temperature dependence of the biological rates via the Boltzmann‐Arrhenius function, which does not capture the non‐linear dependence of most biological rates on temperature (Uszko et al. 2017), and do not consider possible differences in thermal sensitivity between taxa (see Thunell et al. (2021)) for an exception or thermal mismatches (Álvarez‐Codesal et al. 2023; Dee et al. 2020; Meehan and Lindo 2023). In particular, the joint effects of empirically relevant unimodal temperature‐dependent vital rates, thermal mismatches and TSRs across trophic levels have not been investigated.

To address these knowledge gaps, we use a model of a tri‐trophic food chain with size‐ and temperature‐dependent vital rates and trophic interactions to investigate how temperature and size dependence of processes determining consumer life history (growth, development and reproduction), predator functional response and predator metabolic loss contribute to community structure and alternative stable states along gradients of temperature and habitat productivity, and ask whether TSRs in consumers (size at maturation and maximum size) or predators (predation vulnerability size threshold) lead to different outcomes. Finally, we examine how thermal niche mismatches modify the direct kinetic effects of temperature and TSRs on community structure across environmental gradients.

We expect that (1) the temperature dependence of biological processes and TSRs have different consequences for community structure due to their inherently non‐linear (biological processes) and (assumed or estimated) linear relationships (TSRs) with temperature and the different magnitudes of temperature dependence; (2) direct kinetic effects of temperature, rather than TSRs, dominate temperature effects on community structure across temperature and habitat productivity gradients (similar to Bazin et al. (2024)), with processes affecting energy intake by individuals having the strongest effect (as in Lindmark et al. (2022); Reum et al. (2024)); and (3) all else being equal, thermal niche mismatches of interacting species alter our predictions of community transitions across environmental gradients (Dee et al. 2020) due to changes in the consumer‐resource energetic balance (as in Álvarez‐Codesal et al. 2023).

## 2 Methods

### 2.1 Community Model and Temperature Dependent Processes

**FIGURE 1:**
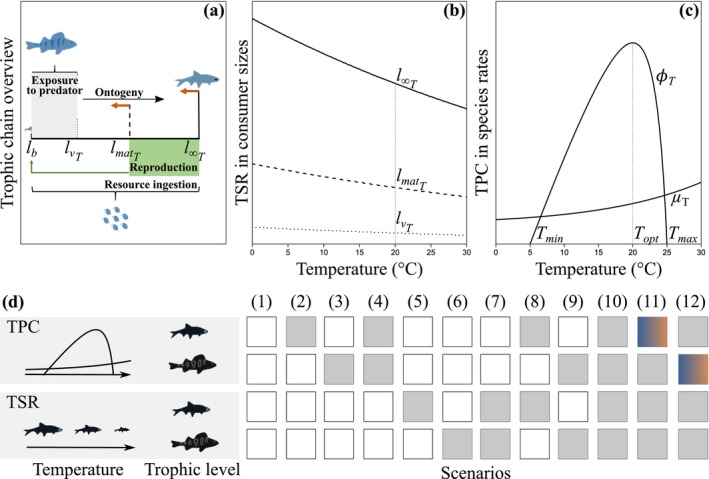
Overview of the model. (a) Summary of the main processes driving the tri‐trophic chain dynamics: Consumer individuals are born at length $l_{b}$, mature at length $l_{{\mathrm{mat}}_{T}}$ and can reach maximum length $l_{\infty_{T}}$ under unlimited food conditions. They feed on the resource following a Holling type II functional response that scales with $l^{2}$ (Equation 1, Table 1) and follow a von‐Bertalanffy growth curve (Equation 2). Adult reproductive investment scales proportionally to $l^{2}$ (Equation 3). Consumer mortality rate decreases with size as in many other aquatic taxa (Pauly 1980) and consists of predation mortality of juvenile consumers smaller than $l_{v_{T}}$ and a constant natural mortality (Equation 4). Top predators feed on vulnerable juvenile consumers following a Holling type II functional response (Equation 5). We use parameters derived for perch ( *Perca fluviatilis* , top predator), European roach ( *Rutilus rutilus* , consumer) and cladocerans (*Daphnia* sp., resource) to parameterise the model (Equations 6–9, Table 1; de Roos and Persson (2002)) and assume that these parameters pertain to the environmental temperature of 20°C (b and c); see Text S1 for details. Orange arrows indicate possible size reductions under warming that influence consumer life history. (b) TSR in consumers can affect their vulnerability to predation ($l_{v_{T}}$, dotted line), size at maturation ($l_{{\mathrm{mat}}_{T}}$, dashed line) and maximum size ($l_{\infty_{T}}$, solid line). (c) TPCs characterising vital rates of consumers and predators. (d) Summary of all scenarios of the effects of TPCs and TSR in consumer and/or predator traits. Grey boxes indicate temperature dependence in the given species and processes/traits, while colour gradient indicates species with shifted thermal niche.

We extend the physiologically structured population model of a tri‐trophic chain developed by de Roos and Persson (2002) to include temperature‐dependent biological rates and TSR in the consumers and top predators. Following de Roos and Persson (2002), we assume that the community is composed of unstructured top predator and basal resource populations and a size‐structured population of consumers characterised by their length *l* (Figure 1a) and use the same dynamic budget model for the consumer population (Text S1). Three different community structures are possible (resource only, consumer‐resource and predator‐consumer‐resource) and an emergent Allee effect with two alternative stable states, consumer‐resource and trophic chain equilibria, occurs at intermediate productivity levels in this model (de Roos and Persson 2002).

We explore the effects of temperature on community structure through (1) TSR that modifies one or more key size thresholds in the consumer species; (2) direct kinetic effects of temperature on the ingestion, growth, and birth rates of the consumer and on the functional response and biomass loss of the predator (hereafter referred to as consumer and top predator ‘vital rates’); and (3) thermal niche mismatches in the vital rates.

We apply the TSR as a reduction of 5%°C^−1^ in consumer size thresholds including the length $l_{v}$ below which juveniles are vulnerable to predation, maturation length *l*_mat_, and/or asymptotic length *l*_∞_ (Figure 1b, Equation 10b), based on the estimated mass‐specific mean reduction under warming for aquatic ectotherms (Coghlan et al. 2024; Forster, Hirst, and Atkinson 2012) and length‐weight allometry of $w\sim l^{3}$ (Text S1). While TSR applied to *l*_mat_ and/or *l*_∞_ mimics reduction in consumer size with warming, TSR applied to predation size threshold $l_{v}$ mimics reduction in predator size with warming. This allows us to explore the consequences of predator–prey size mismatches (Sentis et al. 2024).

Most vital rates can be approximated by unimodal TPCs that are maximised at an intermediate temperature and drop sharply toward the upper thermal limit (Huey and Kingsolver 1989). This is because moderate warming enhances vital rates, but metabolic demand typically increases faster than food intake with increasing temperature (Fussmann et al. 2014; Rall et al. 2010). We applied a Rosso function (Rosso et al. 1995) to the respective proportionality constants (Equation 11b) as in Mallet et al. (1999) and Smalås et al. (2020) to model left‐skewed, non‐linear TPCs characterising consumer ingestion, growth and birth rates, and predator functional response (Figure 1c, Equations 1–5). These rates are maximised at optimal temperature *T*_opt_ and decrease as temperature deviates from *T*_opt_ until reaching zero at thermal boundaries *T*_min_ and *T*_max_. We use *T*_min_ = 5°C, *T*_opt_ = 20°C and *T*_max_ = 25°C for both species in the analyses without thermal niche mismatches (see below). Finally, we model temperature‐dependent consumer mortality rate and predator metabolic loss rate by an exponential increase with temperature (Figure 1c, Equation 12b) as in Uszko et al. (2017).

### 2.2 Analyses

We first explore 10 different scenarios combining temperature‐(in)dependent consumer and predator sizes and vital rates (Equations 10a–12a and 10b–12b, respectively) to disentangle the different effects of temperature on community structure. In the baseline Scenario 1, consumer size thresholds and all vital rates are temperature independent. Scenarios 2–10 implement various combinations of temperature‐dependent vital rates and TSR in the consumer, predator or both species. Scenarios 2–4 explore the direct kinetic effects of temperature in the vital rates of (2) consumers, (3) predators and (4) both consumers and predators. Scenarios 5–7 explore the effects of TSR in (5) consumer sizes (*l*_mat_ and *l*_∞_), (6) predator size ($l_{v}$), and (7) both consumer and predator sizes. Scenarios 8–10 explore the combined effects of temperature in the vital rates and sizes of (8) consumers, (9) predators, (10) both consumers and predators. That is, Scenarios 2, 5 and 8 focus on the temperature effects on consumers alone, Scenarios 3, 6 and 9 focus on the temperature effects on predators alone, while Scenarios 4, 7 and 10 consider that temperature affects both populations.

**FIGURE 2:**
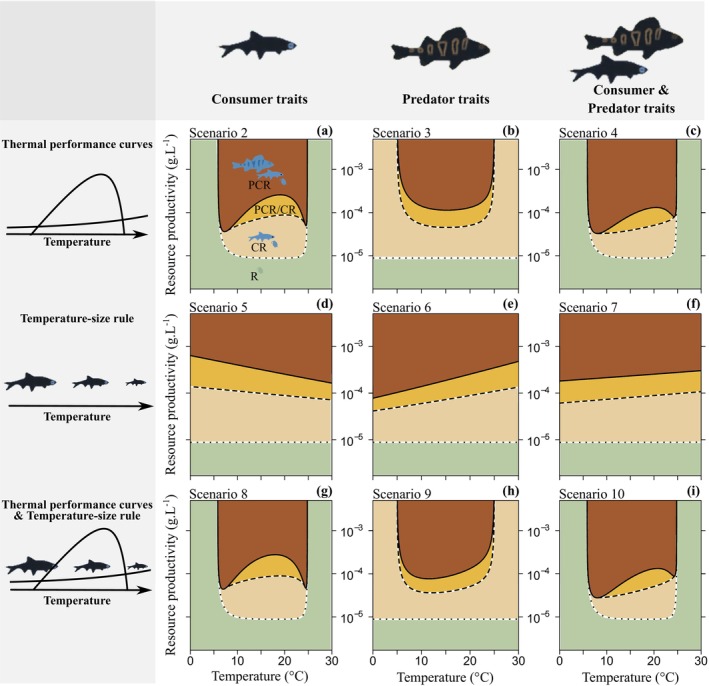
Effects of temperature‐dependent vital rates and TSR on community structure along gradients of habitat productivity and temperature. Each panel shows one scenario: (all panels) convergence of all scenarios results at 20°C when temperature‐(in)dependent parameters are assumed equals (no TSR and species vital rates at their optimum at *T* = *T*_opt_ = 20°C, Scenario 1), (a–c) temperature‐dependent rates only (Scenarios 2–4), (d–f) TSR only (Scenarios 5–7), (g‐i) TSR and temperature‐dependent rates (Scenarios 8–10) in either (a, d, g) consumer traits only, (b, e, h) predator traits only, or in (c, f, i) both consumer and predator traits. Temperature‐dependent rates include (a, c, g, i) consumer growth, ingestion, birth rates, and mortality rate, and (b–c, h–i) predator functional response and metabolic loss rate. TSR implemented in (d, f–g, i) consumer maturation size *l*_mat_ and maximum size *l*_*∞*_, and (e–f, h–i) maximum size *l*_*v*_ exposed to predation. Colours refer to community structure: Resource‐only equilibrium (light green), consumer‐resource equilibrium (CR, ochre), trophic chain equilibrium (PCR, dark red), and alternative stable state (PCR/CR, orange). Dotted lines = consumer invasion threshold; solid line = top predator invasion threshold; dashed line = predator persistence threshold.

Some of these scenarios assume multiple temperature‐dependent parameters (e.g. simultaneous TSR in *l*_mat_ and *l*_∞_ in consumers). We also explore situations in which we consider one temperature‐dependent process or parameter at a time (Figures S3 and S4) or select multiple temperature‐dependent processes (Figure S5) similar to Lindmark et al. (2022) and Reum et al. (2024). This allows us to identify species and processes with the highest effect on community structure along the environmental gradients. We focus on the temperature‐dependent productivity threshold required for consumer establishment (*K*_C_(*T*), dotted lines in Figure 2) and the temperature‐dependent productivity thresholds required for predator establishment and persistence (*K*_P_(*T*) and *K*_A_(*T*), solid and dashed lines in Figure 2) as measures of community transitions, and complement these results by temperature dependence of key individual‐ and population‐level characteristics.

Finally, Scenarios 11 and 12 investigate how mismatches in predator and consumer thermal niche affect the community structure. Using Scenario 10 as a baseline (with all temperature‐dependent vital rates and TSRs), we simulate the consequences of thermal niche shifts of species *j* relative to focal species *i* while maintaining the thermal niche of species *i* (i.e., ~5°C–25°C) for community structure at 13°C (*T* < *T*_opt_) and 20°C (*T* = *T*_opt_). We calculate thermal niche mismatch $∆{\mathrm{TPC}}_{\mathrm{ji}}$ (Equation 13, Table 2) as a shift in the thermal boundaries and optimum temperature of species *j* relative to species *i*. Vital rates of the focal species *j* are thus driven by its shifted thermal niche and environmental temperature, while those of species *i* depend only on environmental temperature. That is, the focal species is either a predator facing a ‘warm‐adapted’ or ‘cold‐adapted’ consumer ($∆{\mathrm{TPC}}_{\mathrm{CP}}$ in Scenario 11, with $∆{\mathrm{TPC}}_{\mathrm{CP}}<0^{{}^{\circ}}C$ and $∆{\mathrm{TPC}}_{\mathrm{CP}}>0^{{}^{\circ}}C$, respectively), or a consumer facing a ‘warm‐adapted’ or ‘cold‐adapted’ predator ($∆{\mathrm{TPC}}_{\mathrm{PC}}$ in Scenario 12, with $∆{\mathrm{TPC}}_{\mathrm{PC}}<0^{{}^{\circ}}C$ and $∆{\mathrm{TPC}}_{\mathrm{PC}}>0^{{}^{\circ}}C$, respectively).

We solve numerically the set of partial and ordinary differential equations describing the tri‐trophic food chain dynamics (Equations 6–9, Table 1) for each scenario, using the package *PSPMAnalysis* version 0.3.9 (de Roos 2021) in the R software version 4.3.2 (R Core Team 2023) to track the system equilibria and detect the critical productivity thresholds *K*_C_(*T*), *K*_P_(*T*) and *K*_A_(*T*).

**TABLE 1:** Equations describing the vital rates of consumers and predators (Equations 1–5) and the state dynamics of tri‐trophic model (Equations 6–9) illustrated in Figure 1a. Temperature‐dependent constants $\Phi_{T}$ of vital rates (Φ = I, B, G or f) and size thresholds ${l_{i}}_{T}$ (*i = v, mat* and *∞*) are described in Table 2. All parameters mare described in Table 3.

Population vital rates	Equation	Eq. no.	State dynamics	Equations	Eq. no.
Ingestion rate $I\left(l,R\right)$	$I\left(\;l,R\right)=I_{T}\;l^{2}\frac{R}{R_{h}+R}$	(1)	Basal resource dynamics	$\frac{\mathrm{dR}}{\mathrm{dt}}=\rho \left(K-R\right)-\int_{l_{b}}^{l_{\infty_{T}}}I\left(l,R\right)c\left(t,l\right)\mathrm{dl}$	(6)
Somatic growth rate $G\left(l,R\right)$	$G\left(\;l,R\right)=G_{T}\left(l_{\infty_{T}}\frac{R}{R_{h}+R}-l\right)$	(2)	Consumer population size‐distribution dynamics	$\frac{\partial c\left(t,l\right)}{\partial t}+\frac{\partial g\left(l,R,T\right)c\left(t,l\right)}{\partial l}=-\mu \left(l,C_{v},P\right)c\left(t,l\right)$ $c\left(t,l_{b}\right)=\int_{l_{{\mathrm{mat}}_{T}}}^{l_{\infty_{T}}}B\left(l,R\right)c\left(t,l\right)\mathrm{dl}$	(7)
Per‐capita birth rate $B\left(l,R\right)$	$B\left(l,R\right)=\left\{\begin{array}{c} 0,\;\text{if}\;l\leq l_{{\mathrm{mat}}_{T}}\\ B_{T}l^{2}\frac{R\;}{R_{h}+R},\;\text{if}\;l>l_{{\mathrm{mat}}_{T}} \end{array}\right.$	(3)			
Mortality rate $\mu \left(l,C_{v},P\right)$	$\mu \left(l,C_{v},P\right)$ $=\left\{\begin{array}{c} \mu_{T_{C}}+f\left(C_{v}\right)P,\;\text{if}\;l\leq l_{v_{T}}\\ \mu_{T_{C}},\;\text{if}\;l>l_{v_{T}} \end{array}\right.$	(4)	Biomass of vulnerable consumers	$C_{v}=\int_{l_{b}}^{l_{v_{T}}}{\mathrm{\omega l}}^{3}c\left(t,l\right)\mathrm{dl}$	(8)
Functional response $f\left(C_{v}\right)$	$f\left(C_{v}\right)=f_{T}*\frac{aC_{v}}{1+\mathrm{ah}C_{v}}$	(5)	Predator biomass dynamics	$\frac{\mathrm{dP}}{\mathrm{dt}}=\left(\epsilon \;f\left(C_{v}\right)-\mu_{T_{P}}\right)P$	(9)

**TABLE 2:** Temperature dependence of consumer and predator traits and vital rates. Temperature‐dependent and temperature‐independent variants of the same parameter or rate always in the same row. Symbols for size thresholds, proportionality constants and mortality rates as in Table 1. $\Phi_{T}=0$ for $T\leq T_{\min_{j}}$ and $T\geq T_{\max_{j}}$ in Equation (11b).

Subject	Temperature‐independent traits and rates	Eq. no.	Temperature‐dependent traits and rates	Eq. no.
Size thresholds $\;l_{i_{T}}$ (*i = v*, *mat* or ∞)	$l_{i_{T}}=l_{i}$	(10a)	$l_{i_{T}}=l_{i}e^{\frac{\beta \left(T-20\right)}{3}}$	(10b)
Proportionality constants $\Phi_{T}$ (Φ = *I*, *B*, *G* or *f; j = C* or *P*)	$\Phi_{T}=\Phi_{T_{\mathrm{opt}}}$	(11a)	$\Phi_{T}=\Phi_{T_{\mathrm{opt}}}\;\frac{\left(T-T_{\min_{j}}\right)\left(T-T_{\max_{j}}\right)}{\left(T-T_{\min_{j}}\right)\left(T-T_{\max_{j}}\right)-{\left(T-T_{{\mathrm{opt}}_{j}}\right)}^{2}}$	(11b)
Background mortality rate and biomass loss rate $\mu_{T_{j}}$ (*j = C* or *P*)	$\mu_{T_{j}}=2\mu_{0}$	(12a)	$\mu_{T_{j}}=\mu_{0}\left(1+e^{-\frac{E\left(\left(T_{{\mathrm{opt}}_{j}}+T_{0}\right)-\left(T+T_{0}\right)\right)}{k\left(T+T_{0}\right)\left(T_{{\mathrm{opt}}_{j}}+T_{0}\right)}}\right)$	(12b)
Species thermal niche mismatch $∆{\mathrm{TPC}}_{\mathrm{ji}}$ (*k* = *min*, *max*, and *opt*; *i* = *C* or *P*, *j* = *C* or *P*, *i* ≠ *j*)	$∆{\mathrm{TPC}}_{\mathrm{ji}}=T_{k_{j}}-T_{k_{i}}$ $T_{k_{j}}=∆{\mathrm{TPC}}_{\mathrm{ji}}+T_{k_{i}}$			(13)

**TABLE 3:** Model parameters.

Subject	Description	Symbol	Default value	Unit
Environment	Temperature	T	20	°C
	Conversion factor from°C to Kelvin	$T_{0}$	273.15	K
	Boltzmann constant	k	8.617 × 10^−5^	eV·K^−1^
Resource	Carrying capacity	K	5 × 10^−4^	g·L^−1^
	Renewal rate	ρ	0.1	day^−1^
Consumer	Length‐weight allometric coefficient	ω	9 × 10^−6^	g·mm^−3^
	Length at birth	$l_{b}$	7	mm
	Predation vulnerability threshold	$l_{v}$	27	mm
	Length at maturation	$l_{\mathrm{mat}}$	110	mm
	Asymptotic length	$l_{\infty }$	300	mm
	Ingestion rate coefficient at optimal temperature $T_{\mathrm{opt}}$	$I_{T_{\mathrm{opt}}}$	10^−4^	g·day^−1^·mm^−2^
	Half‐saturation constant	$R_{h}$	1.5 × 10^−5^	g·L^−1^
	von Bertalanffy growth rate at optimal temperature $T_{\mathrm{opt}}$	$G_{T_{\mathrm{opt}}}$	6 × 10^−3^	day^−1^
	Birth rate coefficient at optimal temperature $T_{\mathrm{opt}}$	$B_{T_{\mathrm{opt}}}$	3 × 10^−3^	day^−1^·mm^−2^
Predator	Attack rate	a	5000	L·day^−1^
	Handling time	h	0.1	day·g^−1^
	Functional response constant at optimal temperature $T_{\mathrm{opt}}$	$f_{T_{\mathrm{opt}}}$	1	Dimensionless
	Food conversion efficiency	ϵ	0.5	Dimensionless
Consumer/predator	Lower temperature threshold for vital rates	$T_{\min}$	5	°C
	Optimal temperature for vital rates	$T_{\mathrm{opt}}$	20	°C
	Upper temperature threshold for vital rates	$T_{\max}$	25	°C
	Scaling constant for mortality rate and biomass loss rate	$\mu_{0}$	5 × 10^−3^	day^−1^
	Activation energy of mortality rate and metabolic loss rate	a	−0.55	eV·K^−1^
	TSR slope	β	−0.05	(°C)^−1^
Individual state/population‐level	Consumer length	l	—	mm
	Population density of consumers	c	—	mm^−1^.L^−1^
	Resource biomass	R	—	g.L^−1^
	Top predator biomass	P	—	g.L^−1^

## 3 Results

**FIGURE 3:**
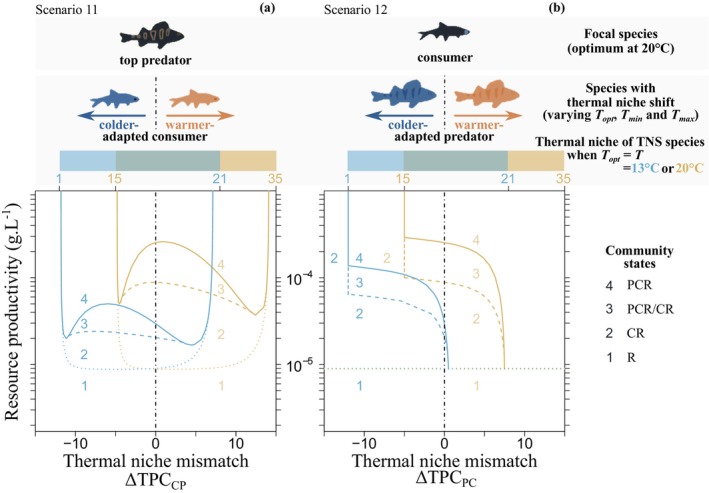
Effects of mismatch between species thermal niches $∆{\mathrm{TPC}}_{\mathrm{ji}}$ on community structure along the habitat productivity gradient at two environmental temperatures ($T=13^{{}^{\circ}}C$ and 20°C). Community transitions highlighted for the thermal niche shift of (a) consumer and (b) predator at $T=13^{{}^{\circ}}C$ and $T=20^{{}^{\circ}}C$. The thermal niche mismatch $∆{\mathrm{TPC}}_{\mathrm{ji}}$ indicates when the thermal niche of species *j* is identical to the focal species niche *i* with optimum temperature $T_{{\mathrm{opt}}_{i}}=20^{{}^{\circ}}C$ as in Scenario 10 (Figure 2i, when $∆{\mathrm{TPC}}_{\mathrm{ji}}=0$) or shifts toward colder ($∆{\mathrm{TPC}}_{\mathrm{ji}}<0$) or warmer temperatures ($∆{\mathrm{TPC}}_{\mathrm{ji}}>0$). Community transitions are numbered from 1 to 4 (1 = R = resource, 2 = CR = consumer‐resource, 3 = PCR/CR = two alternative stable states, 4 = PCR = trophic chain), line types as in Figure 2, colour codes environmental temperature (light blue = 13°C, gold = 20°C). TNS species = species *j* with shifted thermal niche.

We briefly summarise our key findings before detailing them below. All scenarios without the thermal mismatches yield the same results at the optimum temperature *T* = 20°C, when all temperature‐dependent rates are assumed equal to temperature‐independent rates (Scenario 1, Figure S1; *T* = 20°C in Scenarios 2–10, Figure 2; see also de Roos and Persson (2002)). The effects of temperature and habitat productivity on community structure vary markedly between Scenarios 2–10 (Figure 2, see Text S2 for details). Overall, the largest differences in temperature‐dependent productivity thresholds *K*_C_(*T*), *K*_P_(*T*) and *K*_A_(*T*) along the temperature gradient occur between temperature‐dependent vital rates and TSRs. This is because the different shapes and magnitudes of temperature dependence of vital rates (Scenarios 2–4, Figure 2a–c) and TSRs (Scenarios 5–7, Figure 2d–f) strongly affect consumer life histories (Figures S6 and S7) and hence modulate the absolute and relative biomasses of the three consumer stages (Figures S7 and S8; see Text S3 for details). The combined effects of temperature on vital rates and TSRs (Scenarios 8–10, Figure 2g–i) lead to similar responses of community structure along the environmental gradients as in Scenarios 2–4 due to the stronger impact of temperature‐dependent consumer vital rates on their life histories relative to TSRs. Mismatches in species thermal niches further alter community transitions along the environmental gradients, and the effect depends on the species driving the mismatch (Scenarios 11 and 12, Figure 3).

### 3.1 Effects of Temperature on Community Structure Mediated by TPCs

Temperature‐dependent vital rates characterised by unimodal TPCs (Scenarios 2–4, Figure 2a–c) lead to a concave‐up temperature dependence of the productivity thresholds required for the establishment and persistence of the consumer in Scenarios 2 and 4 (*K*_C_(*T*), dotted line in Figure 2a,c) and of the predator in Scenario 3 (*K*_P_(*T*) and *K*_A_(*T*), solid and dashed lines in Figure 2b). The thresholds are almost constant between ~10°C–20°C and increase rapidly when temperature approaches the lower and upper thermal limits (5°C and 25°C). Moreover, temperature‐dependent consumer vital rates lead to highly nonlinear temperature dependence of the predator thresholds *K*_P_(*T*) and *K*_A_(*T*) in Scenarios 2 and 4 (Figure 2a,c). Interestingly, these thresholds are lowest at temperatures close to the lower and upper limits of the consumer thermal niche and highest near the thermal optimum of both species when only consumer rates depend on temperature (Scenario 2, Figure 2a).

A comparison of the effects of temperature‐dependent consumer vital rates (Figure S3a–d) reveals that community transitions in Scenarios 2 and 4 (Figure 2a,c) are primarily driven by temperature‐dependent consumer growth rate (Figure S3c) and ingestion rate (Figure S3a). Accounting for the temperature dependence of predator vital rates (Figure 3e,f) shows that community transitions in Scenario 3 with temperature‐independent consumer vital rates (Figure 2b) are driven by lower predation efficiency (Figure S3e) and that increasing predator biomass loss rate with warming (Figure S3f) causes a higher propensity to collapse near its upper thermal limit in Scenario 4 with temperature‐dependent consumer and predator vital rates (Figure 2c) compared to Scenario 2 (Figure 2a).

### 3.2 Effects of Temperature on Community Structure Mediated by TSR

The effects of temperature on community structure, mediated through reduction of consumer and predator size thresholds (Scenarios 5–7), are simpler than those induced by the temperature‐dependent vital rates. Assuming no thermal limits for either species, the different productivity thresholds for species invasions and persistence depend linearly on temperature (Figure 2d–f).

TSR in all consumer size traits does not alter the productivity threshold *K*_C_(*T*) required for its invasion (Scenarios 5 and 7, dotted lines in Figure 2d–f). This results from compensatory effects of TSR in maturation size and in asymptotic size (Figure S4): warming‐induced reduction of maturation size leads to lower productivity threshold *K*_C_(*T*) (Figure S4b), while reduction of asymptotic size has the opposite effect (Figure S4c). TSR in both maturation and asymptotic size of the consumer also lowers the productivity thresholds for predator invasion *K*_P_(*T*) and persistence *K*_A_(*T*) at higher temperatures (Figures 2d and S4d) as the relative biomass of adults and vulnerable juvenile consumers increases (Figure S8q).

TSR limited to predator (Scenario 6) leads to higher *K*_P_(*T*) and *K*_A_(*T*) thresholds with warming (Figures 2e and S4a) due to lower predation vulnerability threshold $l_{v}$ and hence less prey for the predator. TSR in both consumer and predator traits (Scenario 7) also leads to increasing thresholds *K*_P_(*T*) and *K*_A_(*T*) with warming, but the increase is slower than in Scenario 6 (Figure 2f vs. Figure 2e). This is because the effect of TSR in consumer maturation size *l*_mat_ offsets the effect of TSR in $l_{v}$ (Figure S4e vs. S4g). On the contrary, the combination of TSR in $l_{v}$ and *l*_∞_ leads to an even steeper increase of the *K*_P_(*T*) and *K*_A_(*T*) thresholds under warming (Figure S4f vs. S4g) and the relative biomass of juvenile consumers (Figure S8u vs. S8w).

### 3.3 Combined Effects of Temperature on Community Structure

The combined effects of temperature mediated by TPCs and TSR on community structure (Scenarios 8–10, Figure 2g–i) are very similar to those mediated only by TPCs (Scenarios 2–4, Figure 2a–c), especially when only consumer traits are considered (Figure 2g vs. Figure 2a, Figure 2i vs. Figure 2c). TSR shifts the productivity thresholds for predator invasion and persistence *K*_P_(*T*) and *K*_A_(*T*) toward lower temperatures when temperature affects only the predator (Scenario 9, Figure 2h vs. Figure 2b). This means that the effects of temperature on community structure arise primarily through the varying consumer vital rates, especially ingestion and somatic growth (see above; Figure S3a,c). In particular, faster consumer growth increases the propensity of predator collapse across most of its thermal niche due to its higher demands on productivity required for its establishment (i.e., increasing *K*_P_(*T*) between ~10°C–20°C, Figure 2a,c,g,i).

Interestingly, the combined effects of temperature on the consumer size structure are limited when predators are absent, and they do not differ much among the different types of TSR and the assumption of temperature‐(in)dependent vital rates (Figure S8a–l). The minor effects of different types of TSR come from their limited effects on minimum resource requirements for the consumer population (Figure S6c,d) and relatively small changes in the population regulatory processes (i.e., population growth and birth rates, Figures S6e,f and S7a,c,e). This contrasts with strong effects of temperature on the consumer size structure and much larger differences in the relative proportion of juveniles between the different types of TSR with or without concurrent inclusion of temperature‐dependent vital rates when the consumers are predated (Figure S8m–x).

The emergent Allee effect vanishes when both species are near their thermal limits (i.e., for *T* < 10°C and > ~23°C–24°C), marked by an increasing dominance of the consumer population by non‐vulnerable juveniles (Figures S7lrx and S8rx). Additional scenarios that implement the combination of TSR and TPCs differently in both species confirm that the community transitions along the gradients of temperature and habitat productivity depend primarily on the temperature dependence of consumer vital rates, and to a lesser extent on predator vital rates (Figure S5).

### 3.4 Effects of Thermal Niche Mismatches on Community Structure

Community responses to warming and increased productivity, mainly the predator invasion and collapse, are sensitive to thermal niche shifts in both populations (Figure 3). When predators face colder‐ or warmer‐adapted consumers (Scenario 11), the trends of community transitions along the gradient of thermal niche mismatch (expressed as the difference between the thermal optimum of the consumer and the predator $∆{\mathrm{TPC}}_{\mathrm{CP}}$) mirror the responses to increasing temperature in Scenario 10 (Figure 3a vs. Figure 2i). For example, the predator cannot persist at its optimal temperature of 20°C when facing either a colder‐adapted consumer with $∆{\mathrm{TPC}}_{\mathrm{CP}}<-4.9^{{}^{\circ}}C$ (with thermal optimum below ca. 14.9°C) or a warmer‐adapted consumer with $∆{\mathrm{TPC}}_{\mathrm{CP}}>14^{{}^{\circ}}C$ (thermal optimum above ca. 34°C; solid orange line in Figure 3a) due to very slow consumer growth. The productivity thresholds *K*_P_(*T*) and *K*_A_(*T*) required for predator establishment and persistence at 20°C are highest when the mismatch is minimal with $∆{\mathrm{TPC}}_{\mathrm{CP}}\sim 0^{{}^{\circ}}C$ (solid and dashed orange lines in Figure 3a) due to a rapid growth and short vulnerability period of the juvenile consumers. The thresholds *K*_*P*_(*T*) and *K*_*A*_(*T*) at the environmental temperature of 13°C have a similar shape but are shifted toward lower $∆{\mathrm{TPC}}_{\mathrm{CP}}$ values and lower productivity levels compared to 20°C (blue vs. orange lines in Figure 3a). This results mainly from differences in consumer growth, lower predator biomass loss and larger size range of vulnerable consumer sizes at 13°C as $l_{v}\left(13^{{}^{\circ}}C\right)>l_{v}\left(20^{{}^{\circ}}C\right)$.

Finally, the trends of community transitions along the gradient of thermal niche mismatch differ when consumers face colder‐ or warmer‐adapted predators (Scenario 12). The productivity thresholds for predator invasion and persistence *K*_*P*_(*T*) and *K*_*A*_(*T*) decrease along the gradient of thermal niche shift $∆{\mathrm{TPC}}_{\mathrm{PC}}$ provided the predator thermal niche window overlaps with the environmental temperature; highly cold‐adapted predators ($∆{\mathrm{TPC}}_{\mathrm{PC}}≲-12^{{}^{\circ}}C$ at 13°C and $∆{\mathrm{TPC}}_{\mathrm{PC}}≲-5^{{}^{\circ}}C$ at 20°C) can never persist (Figure 3b). Colder‐adapted predators ($∆{\mathrm{TPC}}_{\mathrm{PC}}<0^{{}^{\circ}}C$) require higher resource productivity to establish relative to warmer‐adapted predators ($∆{\mathrm{TPC}}_{\mathrm{PC}}>0^{{}^{\circ}}C$), and sufficiently warm‐adapted predators ($∆{\mathrm{TPC}}_{\mathrm{PC}}\left(13^{{}^{\circ}}C\right)>1^{{}^{\circ}}C$ and $∆{\mathrm{TPC}}_{\mathrm{PC}}\left(20^{{}^{\circ}}C\right)>7.5^{{}^{\circ}}C$) can always establish together with the consumers (*K*_P_ = *K*_C_) as the alternative stable states rapidly disappear over a 2°C–3°C range of the predator thermal niche shift at the given habitat productivity (Figure 3b).

## 4 Discussion

Our study integrates multiple responses of ectotherms to warming by considering the direct effects of temperature on vital rates, TSR and thermal niche mismatches between predator and prey, and examining their effects on community structure in a size‐structured tri‐trophic food chain. Consistent with our expectations, we show that the effects of temperature‐dependent vital rates on community structure dominate over TSR effects and that even relatively small thermal niche mismatches between interacting species can strongly influence community structure by altering or facilitating the persistence of top predators. Our results also emphasise the importance of thermal sensitivity of growth and feeding in quantifying the consequences of environmental change in aquatic communities.

### 4.1 Comparing the Effects of TPCs, TSR and Species Interactions on Community Structure

We found that the effects of empirically relevant TSR levels on the structure and stability of the tri‐trophic food chain are smaller than the effects of temperature‐dependent vital rates. A recent experiment investigating the effects of TSR in the top fish predator ( *Oryzias latipes* ) and warming on invertebrate communities also concluded that the observed non‐linear changes in invertebrate density and predator–prey biomass ratios with warming were primarily caused by temperature‐dependent invertebrate feeding (Bazin et al. 2024). Taken together, these results suggest that the role of TSR in the effects of warming on community structure may be elusive. However, we did not consider other potentially important pathways, such as the consequences of TSR for mating systems, dispersal and stressor tolerance (Sentis et al. 2024).

Our results do not imply that the effects of TSR on community structure are negligible. Similar to Osmond et al. (2017) and Sentis, Binzer, and Boukal (2017), we found that TSR modulates the productivity thresholds required for predator establishment and persistence. Our results also highlight an overlooked aspect of TSR, which is usually defined for maturation size (Atkinson 1994; Niu et al. 2023). However, TSR can also change asymptotic size (Bazin et al. 2023), which is much larger than maturation size in many taxa with indeterminate growth including fish (McDowall 1994). The relationship between TSR in maturation size and asymptotic size is influenced by the temperature dependence of reproductive investment, which can be optimised in response to warming (Thunell et al. 2023), suggesting that the magnitude of these two TSR responses may differ. We explored scenarios in which one or both sizes depend on temperature and demonstrated that TSRs in both size thresholds lead to qualitatively different outcomes. While fewer resources are needed to maintain the predator population when consumers mature at smaller sizes under warming and grow to the same asymptotic size, TSR in asymptotic size has the opposite effect (see Text S3).

The top‐down effect exerted by top predator on the consumer population was stronger than the effect of TSR under warming in our model, but it also amplified the effects of warming and TSR on consumer size structure. In the studied system, depredation of vulnerable juveniles leads to an ‘abundance overcompensation’ effect that releases adult consumers from intraspecific competition for resources (Gårdmark et al. 2015; Lindmark et al. 2019). Consequently, the temperature dependence of the juvenile‐to‐adult ratio and its sensitivity to different types of TSR was much more pronounced in the tri‐trophic food chain compared to the consumer‐resource system. These context‐dependent effects of TSR in response to warming can thus be attributed to altered intra‐ and interspecific interactions (De Roos and Persson 2013; Uszko, Huss, and Gårdmark 2022), again highlighting the need for trait‐specific data on TSR (Bazin et al. 2024; Sentis et al. 2024). They also identify tri‐trophic systems with highly size‐structured interactions (see also Lindmark et al. 2019) as the most promising empirical systems to quantify the role of TSR in community responses to future warming.

### 4.2 The Role of Thermal Mismatches of Interacting Species in Community Transitions

We found that thermal mismatches between consumers and top predators can alter community transitions across environmental gradients and prevent a sudden collapse of the top predator population. Recent studies on thermal mismatches in trophic interactions have shown how species‐specific temperature effects on energetic balance modulate predator–prey energetic balance and species interactions strengths (Álvarez‐Codesal et al. 2023). For example, thermal mismatches between prey growth rate and predator feeding efficiency determine predator persistence and stability (Dee et al. 2020).

We extend these studies to size‐structured interactions in a tri‐trophic system. We found that productivity thresholds for predator establishment and persistence are sensitive to the thermal niche of prey, vary strongly with the thermal niche of predator and depend on which of the two species is adapted to higher temperatures. In our model, predators that feed on consumers that are close to their thermal limit were less likely to collapse, and sufficiently warm‐adapted predators could always invade and avoid collapse if the habitat could support the consumer population. We attribute these results to a stronger top‐down pressure on a slower growing prey (as in Meehan and Lindo 2023). These findings confirm other predictions of future successful invasions of warmer‐adapted species (Bellard et al. 2013; Seebens et al. 2021; Sentis, Montoya, and Lurgi 2021) and the ability of warm‐adapted predators to survive at higher temperatures (Thunell et al. 2021).

### 4.3 The Process Specificity of Community Responses to Warming

Many processes contribute to community responses to environmental change, and contributions can differ both qualitatively and quantitatively (Lindmark et al. 2022; Reum et al. 2024; Sentis, Binzer, and Boukal 2017). Our comparison of different model variants, in which only a subset of all possible mechanisms were ‘switched on’, revealed that consumer ingestion and somatic growth rates are the two most influential processes modulating the response of community structure to environmental change. Recent multi‐species size spectra models examining community responses to a given warming scenario found that mean species size and total spawning stock biomass (Reum et al. 2024) or size‐at‐age (Lindmark et al. 2022) increase substantially when the model incorporates temperature‐dependent intake rates of all species. Our results demonstrate that the temperature‐dependent intake rate also influences changes in community structure along environmental gradients and that its effect is species‐dependent. In our model, the temperature dependence of intake rate or growth rate of the consumer alters its exposure to predation and hence the amount of energy available to predators. We hypothesise that such effects should be common, that is the temperature dependence of intake or growth rates of species with the strongest effects on energy flows between trophic levels should have the strongest effects on community responses to warming. This also means that robust data on the temperature dependence of intake or growth rates are crucial for accurate predictions.

Non‐linear TPCs of vital rates lead to non‐linear temperature dependence of habitat productivity thresholds required for system persistence and stability in a consumer‐resource system (Uszko et al. 2017; our model excluding predators). Moreover, size‐structured interactions between consumers and top predators, characterised by non‐linear TPCs, affect the productivity thresholds required for predator establishment and persistence in our model, with the largest range of habitat productivity separating both productivity thresholds of the top predator at the joint optimal temperature of both species. The propensity for catastrophic collapses of such size‐structured, tri‐trophic food webs is modified by other assumptions: for example, it decreases if the resource carrying capacity does not change with temperature (Lindmark et al. 2019). By considering non‐linear TPCs, we showed that the propensity to collapse increases with future warming if current temperatures are below the species' optimum, but decreases as temperature approaches the species' upper thermal limits. We used a specific functional form of TPCs, but assume that our qualitative conclusions also apply to other functional forms of TPCs such as the Sharpe‐Schoolfield equation (Pawar, Dell, and Savage 2012; Schoolfield, Sharpe, and Magnuson 1981).

Similar to the differential effects of vital rates discussed above, the effects of TSR on the persistence and stability of a simple tri‐trophic food chain vary predictably with the direction and magnitude of TSR and with the trophic level of species exhibiting TSR (Sentis, Binzer, and Boukal 2017). We showed that TSR lead to less abrupt community transitions across the temperature gradient (Scenarios 5–7, Figure 2). We only considered declining body sizes with warming, with the TSR slope corresponding to the average value for aquatic ectotherms (Coghlan et al. 2024; Forster, Hirst, and Atkinson 2012). Coghlan et al. (2024) on warming‐induced changes in body size within marine fish guilds imply that our assumptions would need to be modified for some communities. For example, one might expect a higher predation vulnerability threshold under warming following an increase in piscivorous fish body size as reported by Coghlan et al. (2024), which would consequently affect habitat productivity thresholds required for top predator invasion and persistence.

## 5 Conclusions

Body size and temperature determine many ecological processes from individuals to entire communities (Brown et al. 2004). Using a tri‐trophic food chain model with temperature‐ and size‐dependent interactions, we demonstrate that a detailed understanding of the temperature and size dependence of vital rates and life histories is needed to better predict future community responses to global change, including the propensity for regime shifts. Importantly, we discover that the direct effects of warming and TSRs have very different consequences for community structure and that the importance of TSRs may be limited. On the other hand, relatively small thermal niche mismatches in interacting species can alter community structure even in a simple tri‐trophic food chain. Future studies should thus explore the role of temperature‐ and size‐dependent life histories (Ohlberger et al. 2011) across multiple taxa and trophic levels, including asymmetric thermal responses in interacting populations (Dell, Pawar, and Savage 2014), to understand the responses of more complex food webs to global change.

## Author Contributions

D.S.B. and S.D. designed the study, S.D. performed the analyses. All authors discussed the results and made suggestions for their presentation. S.D. and D.S.B. wrote the first draft of the manuscript, and all co‐authors revised it.

## Conflicts of Interest

The authors declare no conflicts of interest.

### Peer Review

The peer review history for this article is available at https://www.webofscience.com/api/gateway/wos/peer‐review/10.1111/ele.70022.

## Supporting information


Data S1.

## Data Availability

No new data were used in this study. All code required to replicate the results have been deposited in GitHub (https://github.com/Samuel‐Dijoux/2024‐PSPM‐TPC_TSR) and Zenodo (https://doi.org/10.5281/zenodo.10993083).
