# Supplementary material for: Differences in Tri‐Trophic Community Responses to Temperature‐Dependent Vital Rates, Thermal Niche Mismatches and Temperature‐Size Rule
Source: Ecol Lett. 2024 Dec 2;27(11):e70022. doi: 10.1111/ele.70022 (PMC11612537; doi:10.1111/ele.70022)

**Supplementary information for**

**Differences in tri-trophic community responses to temperature-dependent vital rates, thermal niche mismatches and temperature-size rule**

Authors: Samuel Dijoux^1,2,*^, Aslak Smalås^3,4^, Raul Primicerio^3^, David S. Boukal^1,2^

^1.^ Department of Ecosystems Biology, Faculty of Science, University of South Bohemia, České Budějovice, Czech Republic.

^2.^ Czech Academy of Sciences, Biology Centre, Institute of Entomology, České Budějovice, Czech Republic.

^3.^ Department of Arctic and Marine Biology, Faculty of Biosciences, Fisheries and Economy, UiT, The Arctic University of Norway, Tromsø, Norway.

^4.^ SNA-Skandinavisk naturoveråking AS (Scandinavian Nature-monitoring), DNV, Tromsø, Norway.

^*^E-mail addresses: dijous00@prf.jcu.cz (S. Dijoux); dboukal@prf.jcu.cz (D. S. Boukal).

This supplementary material contains the following texts and figures:

[Text S1.](#Text_S1) Dynamic energy budget model of the consumer life history.

[Text S2.](#Text_S2) Community structure along environmental gradients.

[Text S3.](#Text_S3) Effects of temperature on the consumer life histories and population structure.

[Fig. S1.](#Fig_S1) Examples of community transitions along the gradient of habitat productivity.

[Fig. S2.](#Fig_S2) Examples of community transitions along the temperature gradient.

[Fig. S3.](#Fig_S3) Influence of temperature-dependent vital rates on community structure along gradients of temperature and habitat productivity.

[Fig. S4.](#Fig_S4) Influence of TSR on community structure along gradients of temperature and habitat productivity.

[Fig. S5.](#Fig_S5) Influence of temperature-dependent vital rates and TSR on community structure along gradients of temperature and habitat productivity.

[Fig. S6.](#Fig_S6) Effects of warming on consumer life history, minimum resource requirements and population growth rate.

[Fig. S7.](#Fig_S7) Temperature dependence of consumer birth rate and stage-specific biomasses.

[Fig. S8.](#Fig_S8) Cumulative biomass proportions of consumer stages across temperature gradient.

**Text S1. Dynamic energy budget model of the consumer life history**

We use the same dynamic energy budget (DEB) model as in Dijoux & Boukal (2021) and de Roos & Persson (2002) to describe how individual size- and resource-dependent growth and reproduction vary with temperature. This so-called Kooijman-Metz model is a widely used DEB model that falls in the category of kappa-rule models, in which the ingested energy is first invested in maturation, reproduction and somatic growth, before covering the maintenance costs (De Roos *et al.* 1990; Noonburg *et al.* 1998; de Roos & Persson 2002; Smallegange *et al.* 2017).

Consumer life histories are characterized by size-, resource- and temperature-dependent feeding rate, growth rate and fecundity rate and size-, predator- and temperature-dependent mortality rate (Table 1). Individual consumers are born at length $l_{b}$ and remain exposed to predation until reaching length $l_{v}$. They mature when reaching length $l_{mat}$ and continue growing towards the asymptotic length $l_{\infty}$ under unlimited food conditions. We assume that the length at birth *l_b_* is always constant but allow the other size thresholds $l_{v}$, $l_{mat}$ and $l_{\infty}$ to vary with temperature (see the main text for details).

The rate of energy acquisition is assumed proportional to body surface ($\sim l^{2}$), while maintenance is proportional to body weight ($\sim l^{3}$). That is, the ingestion rate $I\left( l,R \right)$ of an individual consumer with length $l$ feeding on the basal resource *R* follows a type II functional response (Eq. 1). Given that maintenance increases faster with body size than the ingestion, individual consumers follow a von Bertalanffy growth curve with resource- and temperature-dependent growth rate $G\left( l,R \right)$ and asymptotic size (Eq. 2). They produce offspring after maturation at a *per* *capita* rate $B\left( l,R \right)$ that is proportional to $l^{2}$ (Eq. 3). For simplicity, we assume that the individuals stop growing and reproducing but do not shrink or use energy reserves to cover maintenance costs when the food intake becomes insufficient. However, these conditions do not occur in a system at equilibrium, which makes them negligible for the purpose of our study; see (de Roos *et al.* 1990) for details. In addition to predation mortality, individual consumers die with the same size-independent background mortality rate $\mu_{C}$ (Eq. 4) that may be temperature-dependent (Eq. 12a and 12b).

Top predators feed indiscriminately on the biomass of vulnerable juvenile consumers $C_{v}$ when present, following a Holling type II functional response $f\left( C_{v} \right)$ (Eq. 5), and face a metabolic mass loss that may be temperature-dependent (Eq. 12a and 12b). We assume constant conversion efficiency $\epsilon$ of ingested prey biomass to predator biomass.

Our assumptions of temperature-dependent maximum ingestion rate, somatic growth rate and per capita birth rate (Eqs 1–3), modelled by the Rosso function (Eq. 11b), and background mortality rate (Eq. 4), characterised by the Boltzmann-Arrhenius dependence on temperature (Eq. 12b) result in an energy budget model of the consumer in which the surplus energy available for growth and reproduction increases with warming until just below the optimal temperature *T_opt_* and then declines rapidly between *T_opt_* and *T_max_*. Moreover, the individual energy budget of consumers is negative at temperatures just above *T_min_* and just below *T_max_*, and the interval of temperatures at which the consumer population can survive therefore does not reach these thermal limits (Fig. 1c). To avoid negative values of the Rosso function at temperatures outside species thermal boundaries (i.e. for *T* < *T_min_* and *T* > *T_max_*), we fix species rates at 0 at those temperatures.

Our model describes a stage-structured, rather than a fully size-structured consumer population, and does not address how a TSR arises from temperature-dependent rates. Various mechanisms can explain TSR (Sentis *et al.* 2024), including the differential thermal sensitivity of growth and developmental rates and differential effects of temperature on vital rates during development (Audzijonyte *et al.* 2022). Instead, we take TSR as given and use it to compare the consequences of the direct kinetic effects of warming, TSR and thermal mismatches on community structure.

We also assume that TSR may arise from constraints on individual life histories that do not necessarily require ecological feedbacks and can therefore be treated as a separate phenomenon, although individual responses to warming and predation risk may be similar, including their consequences for species interactions and community structure (Sentis *et al.* 2024). By separating the effects of temperature-dependent vital rates and TSR, we follow the approach of several other recent modelling studies (Lindmark *et al.* 2022; Reum *et al.* 2024) to understand which processes may play an important role in community responses to warming. Lindmark *et al.* (2022) and Reum *et al.* (2024) focused on different processes that determine energy acquisition and allocation, and switched on and off the temperature dependence of these processes simultaneously in all species. We also investigate situations where we switch the temperature dependence only in one process or in one species. This allows us to identify the critical processes that determine community responses to warming.

**Text S2. Community structure along environmental gradients**

The observed community transitions across the habitat productivity gradient are similar at most temperatures, with the basal resource-only community replaced by the consumer-resource and subsequently by the resource-consumer-predator system as habitat productivity increases, and a range of habitat productivities where the community has two alternative stable states with and without the top predator (Fig. S1).

Community transitions across the temperature gradient can be more diverse than across the habitat productivity gradient, especially when considering the direct effects of warming on species traits at intermediate levels of habitat productivity that often lead to the alternative stable states (Fig. 2). Here, we describe only the community responses to warming in Scenarios 8–10, with habitat productivity fixed at an intermediate level *K* = 10^-4^ g.L^-1^ (Fig. 2g-i). If temperature only affects consumer traits (Scenario 8, Fig. S2a), warming triggers a community transition from a resource-only system at low temperatures (below ~6°C) to a stable tri-trophic food chain once the consumer (blue stars in Fig. S2a) and top predator (black stars in Fig. S2a) can invade and survive in the system. The stable tri-trophic chain becomes bistable between ~10°C and ~23°C (with the potential collapse of the top predator, e.g. when predator mortality increases or when habitat productivity decreases as in Fig. S1) and then becomes stable again between ~23°C and ~25°C. Finally, the system rapidly changes to a resource-only state as the temperature exceeds the consumer thermal limit above ~25°C. The alternative stable states between ~10–23°C include a consumer-resource state (thin lines in Fig. S2a) and a tri-trophic chain (thick lines in Fig. S2a).

When temperature only affects predator traits (Scenario 9, Fig. S2b), warming leads to an abrupt transition from a consumer-resource to tri-trophic chain at ~8°C (black stars in Fig. S2b), which collapses back to a consumer-resource system at ~24°C (red stars in Fig. S2b). A gradual decrease in temperature leads to an abrupt transition from a consumer-resource to tri-trophic chain at ~17°C, which collapses back to a consumer-resource system at ~6°C. That is, the system exhibits two alternative stable states between ~6–8°C and ~17–24°C. The top predator population collapses at ~6°C (i.e. near the lower limit of its thermal performance curve) due to the decline in foraging efficiency and at ~24°C (i.e. near the upper limit of its thermal performance curve) due to its increasing biomass loss rate and the decline in biomass of juvenile consumers exposed to predation, caused by the decline in $l_{v}$ mediated by TSR.

When temperature affects both consumer and predator traits (Scenario 10, Fig. S2c), warming leads to the same sequence of community structure transitions as in Scenario 8 (Fig. S2a), but the tri-trophic chain remains stable for a wider temperature range (~6–16°C) at temperatures below the optimum. In particular, temperature-dependent predator traits lead to lower biomass of vulnerable juvenile consumers for most temperatures where the top predator is present at the given level of habitat productivity (compare Fig. S2bc with Fig. S2a).

**Text S3. Effects of temperature on the consumer life history and population structure**

The effects of temperature mediated by TSR on the life histories and population structure of consumers depend on the measure of body size affected by TSR (Eq. 10b, Table 2). Below, we distinguish between three cases where TSR affects only size at maturation ($l_{mat}$), only asymptotic size ($l_{\infty}$), or both $l_{mat}$ and $l_{\infty}$, and combine these with the absence or presence of TSR in the predator, resulting in temperature-independent and temperature-dependent vulnerable size threshold of juvenile consumers $l_{v}$ (i.e. six cases in total). We illustrate the effects of temperature at an intermediate habitat productivity (*K* = 1 10^-4^ g.L^-1^).

***Effects of temperature on consumers mediated by TSR***

A reduced maturation size $l_{mat}$ leads to earlier maturation (solid line in Fig. S6a) and thus to a longer adult lifespan under warming. This lowers the minimum resource requirement for consumers (solid line in Fig. S6c) and increases the population growth rate (solid line in Fig. S6e) and the birth rate (black line in Fig. S7a) with warming. As a result, the biomass of vulnerable juveniles (black line in Fig. S7g) and adults (black line in Fig. S7s) increase with warming, mainly due to earlier maturation, and the biomass of non-vulnerable juveniles (black line in Fig. S7m) remains almost constant and almost entirely accounts for the consumer biomass (dark grey area, Fig. S8a).

The presence of the top predator at temperatures above ~15°C increases the biomass of vulnerable juveniles through an ‘abundance overcompensation’ (solid red line in Fig. S7g) (Gårdmark *et al.* 2015): Predation of vulnerable juveniles leads to a decrease in the biomass of non-vulnerable juveniles (solid red line in Fig. S7m), which releases adults from intraspecific competition with juveniles (solid red line in Fig. S7s) and significantly increases consumer birth rate (solid red line in Fig. S7a). This in turn increases the biomass of vulnerable juveniles, which compensates for the biomass loss due to predation and lead to a (near) constant, temperature-independent biomass (solid red line in Fig. S7g). The presence of predation thus leads to a reversal of stage dominance within the consumer population under warming (Fig. S8m), from a dominant juvenile stage (almost 60% of total biomass at ~16°C, dark grey area) to a dominant adult stage (up to 80% of total biomass at 30°C, white area).

We observed that predators can survive at temperatures below ~15°C and that the relative biomasses of individual consumer stages vary much less with temperature when we combined the TSRs in the consumer ($l_{mat}$) and the predator ($l_{v}$; dotted red lines in Fig. S7agms), which is mainly due to a decrease in the consumer birth rate (red dotted line in Fig. S7a) and a relatively higher biomass of vulnerable juveniles at lower temperatures (light grey area in Fig. S8s). The changing threshold for vulnerable size and maturation size across the temperature gradient has a balancing effect on the structure of the consumer population, as the dominance of the adults over juveniles varies only between ~55% of the total biomass at the temperature extremes (0°C and 30°C) and ~60% at ~15°C (white area in Fig. S8s), i.e. the effect of temperature on consumer population is much smaller than for the other combinations of temperature-dependent traits and processes (Fig. S8m-r and S8t-x).

The effects of reduced asymptotic size $l_{\infty}$ with warming are opposite to those observed for the warming-induced decrease in maturation size described above, because the declining asymptotic size in our model leads to a slower somatic growth rate at higher temperatures (Table 1, Eq. 2). This leads to delayed maturation (dashed line in Fig. S6a) and an increasing minimum resource requirement of consumers with warming (dashed line in Fig. S6c). Slower individual growth also leads to a lower population growth rate (dashed line in Fig. S6e), birth rate (black line in Fig. S7c) and stage-specific biomass (black lines in Fig. S7iou), but without affecting population structure (Fig. S8c). The increased availability of prey due to its slower growth allows the predator population to survive until its collapse at ~29°C (red line in Fig. S7i), but the predator-induced overcompensation in consumer population decreases with warming as the consumer population shifts from being dominated by adults to a domination by non-vulnerable juveniles (Fig. S8o). The decrease of predator-induced overcompensation effect on consumer population is even steeper when we combine the TSR in consumers ($l_{\infty}$) and predators ($l_{v}$) (Figs. S7iou and S8u): The reduction of the vulnerable size limit of consumers with warming reduces prey availability and the predator population collapses already at ~23°C (dotted red line in Fig. S7i).

The contrasting responses of life history and population structure of consumers when TSR causes lower maturation size ($l_{mat}$) and asymptotic size ($l_{\infty}$; and hence lower somatic growth rate) with warming lead to intermediate and sometimes negligible responses when both $l_{mat}$ and $l_{\infty}$ are affected by TSR. Individuals mature at the same age and have the same minimum resource requirements at all temperatures (dotted lines in Fig. S6ac), while the birth rate of the consumer population decreases slightly with warming (dotted lines in Fig. S6e). Consumer birth rate and the biomasses of vulnerable juveniles and adults increase with warming similarly to the situation in which TSR only affects maturation size (cf. black lines in Fig. S7ekqw and S7agms); only the total biomass of juveniles declines with warming (black line in Fig. S7q). The presence of the top predator above ~15°C and its top-down effects on the consumer birth rate and the biomasses of individual stages are similar to those when TSR only leads to a lower maturation size $l_{mat}$ (cf. solid red lines in Fig. S7ekqw and S7agms, and Fig. S8mq). This means that the consequences of reduced maturation size are greater than those of asymptotic size and individual growth rate when predators are present. Surprisingly, the role of reduced maturation size becomes much smaller when we combine TSR in consumers ($l_{mat}$ and $l_{\infty}$) and predators ($l_{v}$). In this case, the effects of the predator on the consumer population are similar to those when TSR affects only $l_{\infty}$ and $l_{v}$ (cf. dotted red lines in Fig. S7ekqw and S7ciou, and Fig. S8uw).

***Combined effects of temperature on consumers***

The response of consumers to warming at the life history and population levels is dominated by the direct effects of temperature on consumer vital rates, compared to the effects mediated by TSR, and the various TSR mechanisms play only a limited role in the responses. Consumer vital rates (growth, ingestion and birth rate) are maximised at the optimum temperature *T*_opt_ = 20°C and decrease as the temperature moves away from the optimum (Table 2, Eq. 11b). Consumers thus mature fastest and the population growth rate is maximised at or near the optimal temperature irrespective of the TSR mechanism (Fig. S6b and S6f). Interestingly, the minimum resource requirements of the consumer population in our model are sensitive to the TSR mechanisms. The lowest resource requirement occurs near the optimal temperature when TSR affects maturation size $l_{mat}$ (solid and dotted line in Fig. S6d) and at a much lower temperature (~11°C) when TSR affects the asymptotic size *l_∞_* (dashed line in Fig. S6d). We attribute these differences to the interaction between the maximised growth of individuals observed in Fig. S6f and the minimum resource requirements of the consumer population across the temperature gradient relative to the effect of TSR (Fig. S6c). Although we cannot clearly demonstrate this mechanism in empirical data, it shows the importance of ecological feedbacks when considering the role of TSR in the effects of warming on ectotherm populations.

If we also consider the direct effects of temperature on consumers, their per capita birth rate (black line in Fig. S7bdf) and the biomass of vulnerable juveniles (black line in Fig. S7hjl), the total biomass of juveiles (black line in Fig. S7npr) and the biomass of adults (black line in Fig. S7tvx) all show a convex dependence on temperature over most of the thermal range (*T*_min_, *T*_max_) and only decline abruptly when the temperature reaches *T*_min_ or *T*_max_. This means that the consumer population is most strongly limited by the density-dependent feedback near its optimum temperature, characterised by the lowest birth rate across the temperature gradient (Fig. S7bdf) and the lowest biomass of adults and vulnerable juveniles (Fig. S7hjl and Fig. S7tvx). In contrast, the density-dependent feedback becomes weaker at temperatures close to the thermal limits of the consumer and is characterised by peaks in reproduction and relative biomasses of adults and vulnerable juveniles that reduce the relative biomass of non-vulnerable juveniles (Fig. S8bdf).

The overcompensation effect on the consumer population caused by predation strongly impacts its population structure across the temperature gradient (Fig. S8npr); predation on vulnerable juveniles (solid red lines in Fig. S7hjl) reduces the biomass of non-vulnerable juveniles (solid red lines in Fig. S7npr), which releases adults from intraspecific competition with juveniles (solid red lines in Fig. S7tvx vs. S7npr) and leads to increased population birth rates (solid red lines in Fig. S7bdf). This leads to a dominance of adults over most of the temperature gradient, while the juvenile stage dominates again near the thermal limits where predation is least effective (Fig. S8npr). The differences in the effects of mediated by TSR in consumer and predator traits on community structure and predator collapse are much smaller than those described for the effects mediated by TSR in consumer traits alone (solid vs. dotted and dashed vs. dot-dashed red lines in Fig. S7, and Fig. S8npr vs. S8tvx).

**References**

Audzijonyte, A., Jakubavičiūtė, E., Lindmark, M. & Richards, S.A. (2022). Mechanistic Temperature-Size Rule Explanation Should Reconcile Physiological and Mortality Responses to Temperature. *The Biological Bulletin*, 243, 220–238.

De Roos, A.M., Metz, J.A.J., Evers, E. & Leipoldt, A. (1990). A size dependent predator-prey interaction: who pursues whom? *J. Math. Biol.*, 28, 609–643.

Dijoux, S. & Boukal, D.S. (2021). Community structure and collapses in multichannel food webs: Role of consumer body sizes and mesohabitat productivities. *Ecology Letters*, 24, 1607–1618.

Gårdmark, A., Casini, M., Huss, M., van Leeuwen, A., Hjelm, J., Persson, L., *et al.* (2015). Regime shifts in exploited marine food webs: Detecting mechanisms underlying alternative stable states using size-structured community dynamics theory. *Philosophical Transactions of the Royal Society B: Biological Sciences*, 370, 1–10.

Lindmark, M., Audzijonyte, A., Blanchard, J.L. & Gårdmark, A. (2022). Temperature impacts on fish physiology and resource abundance lead to faster growth but smaller fish sizes and yields under warming. *Global Change Biology*, 28, 6239–6253.

Noonburg, E.G., Nisbet, R.M., Mccauley, E., Gurney, W.S.C., Murdoch, W.W. & DE Roos, A.M. (1998). Experimental testing of dynamic energy budget models. *Functional Ecology*, 12, 211–222.

Reum, J.C.P., Woodworth‐Jefcoats, P., Novaglio, C., Forestier, R., Audzijonyte, A., Gårdmark, A., *et al.* (2024). Temperature‐Dependence Assumptions Drive Projected Responses of Diverse Size‐Based Food Webs to Warming. *Earth’s Future*, 12, e2023EF003852.

de Roos, A.M. & Persson, L. (2002). Size-dependent life-history traits promote catastrophic collapses of top predators. *Proceedings of the National Academy of Sciences of the United States of America*, 99, 12907–12912.

Sentis, A., Bazin, S., Boukal, D. & Stoks, R. (2024). Ecological consequences of body size reduction under warming. *Proceedings of the Royal Society B: Biological Sciences*.

Smallegange, I.M., Caswell, H., Toorians, M.E.M. & de Roos, A.M. (2017). Mechanistic description of population dynamics using dynamic energy budget theory incorporated into integral projection models. *Methods in Ecology and Evolution*, 8, 146–154.

**Fig. S1. Examples of community transitions along the gradient of habitat productivity.** (a) Transitions observed at the optimal temperature (*T* = 20°C) in all scenarios (see Fig. 2), and (b) at *T* = 13°C in Scenario 10. Solid lines = stable equilibria, dashed lines = unstable equilibria, dotted vertical lines = threshold resource productivities. Colours as in Fig. 2.


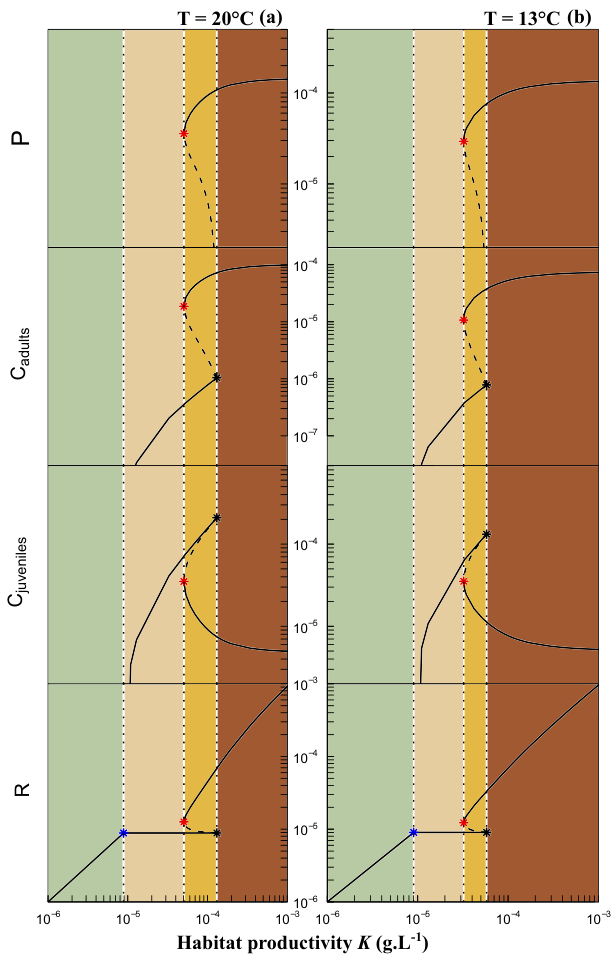


**
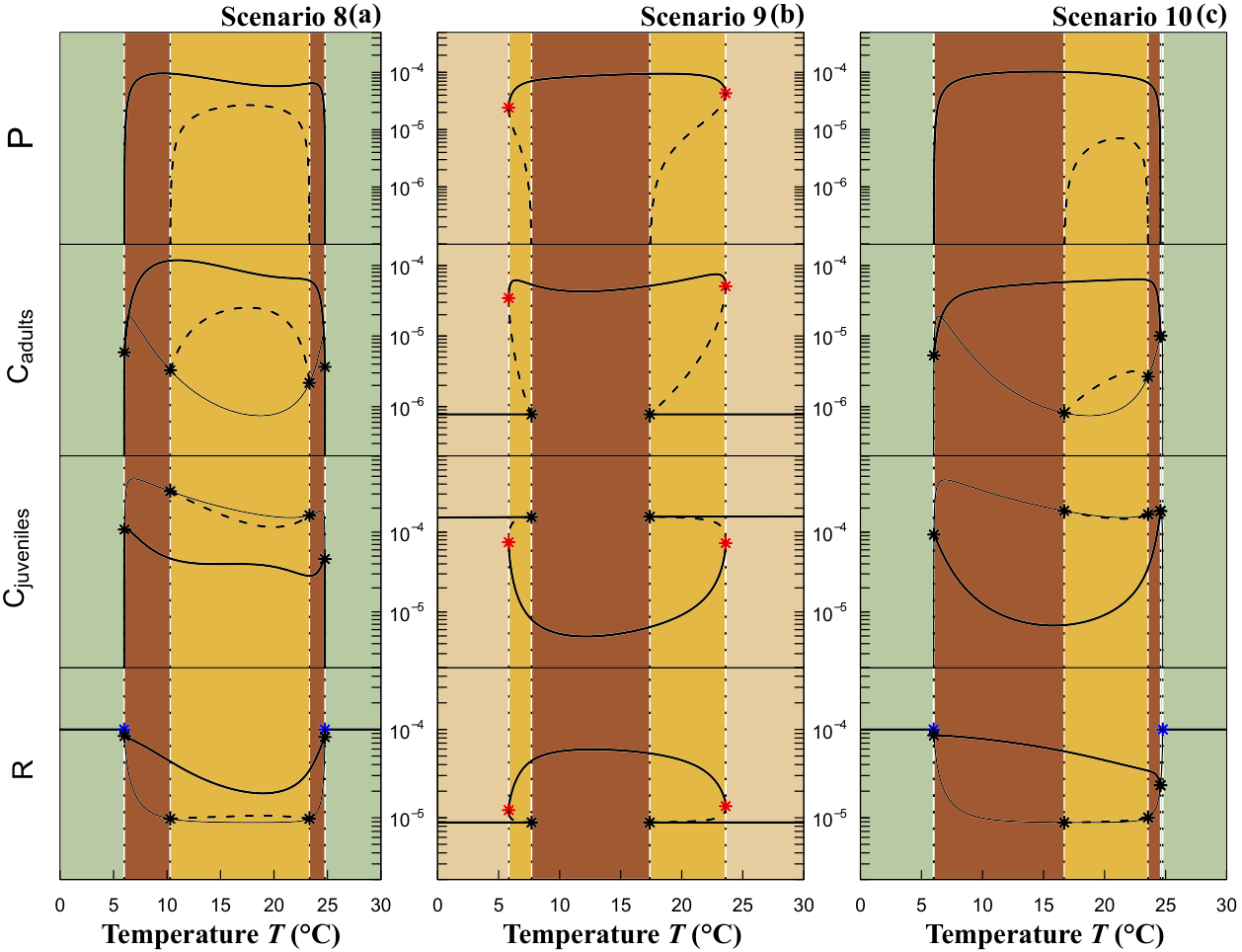
Fig. S2. Examples of community transitions along the temperature gradient.** Transitions at constant habitat productivity (K = 10^-4^ g.L^-1^) with temperature-dependent vital rates and TSR implemented in consumer only (a, Scenario 8), predator only (b, Scenario 9), and in consumer and predator (c, Scenario 10). Solid lines = stable equilibria, dashed lines = unstable equilibria, dotted vertical lines = threshold resource productivities (a) and temperatures (b-c). Thin solid line in (a) and (c) = consumer-resource equilibrium in the absence of predator. Colours as in Fig. 2.

**Fig. S3. Influence of temperature-dependent vital rates on community structure along gradients of temperature and habitat productivity**. TPC limited to one rate at the time in consumer (a-d) and predator (e, f) traits. Colours, line types and community structure as in Fig. 2.

**
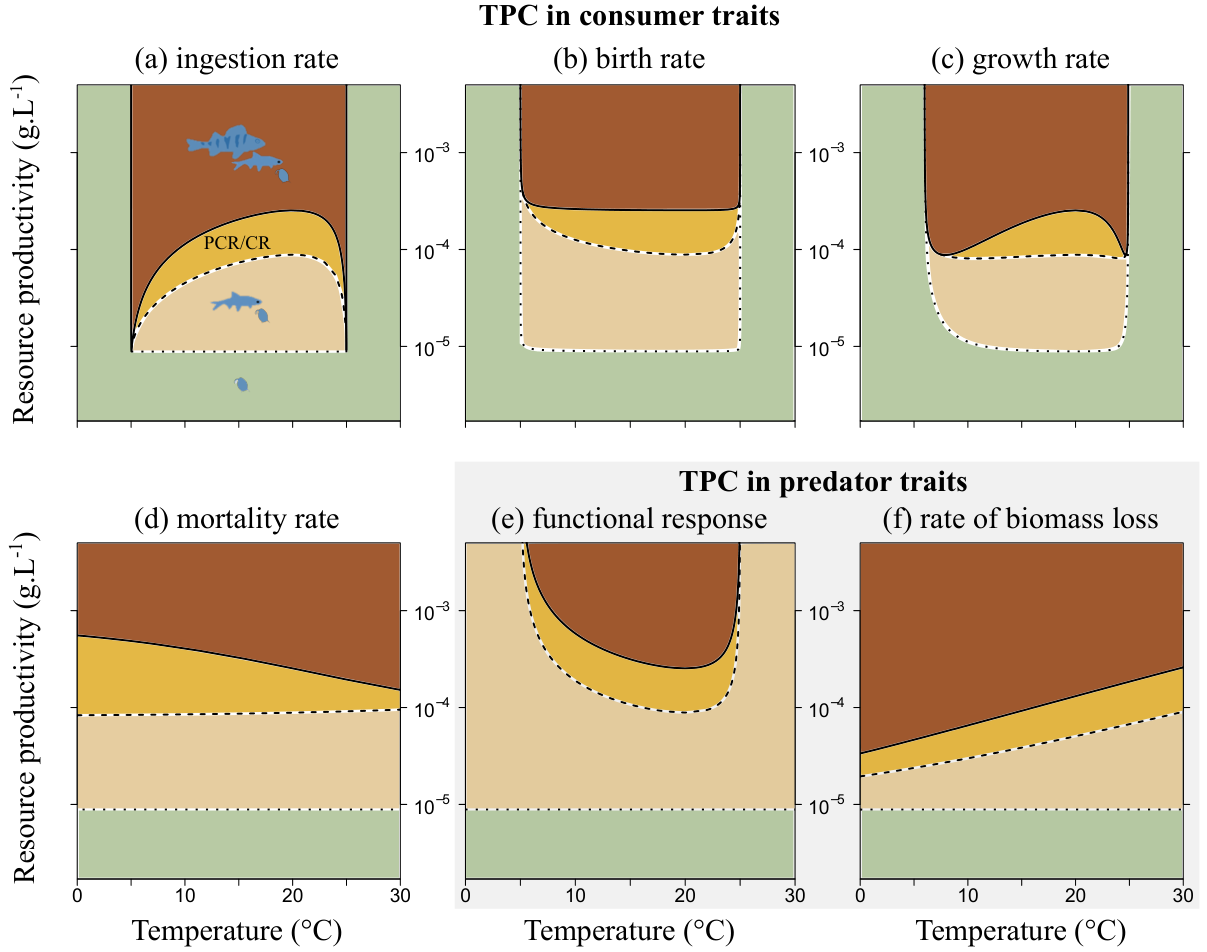
**


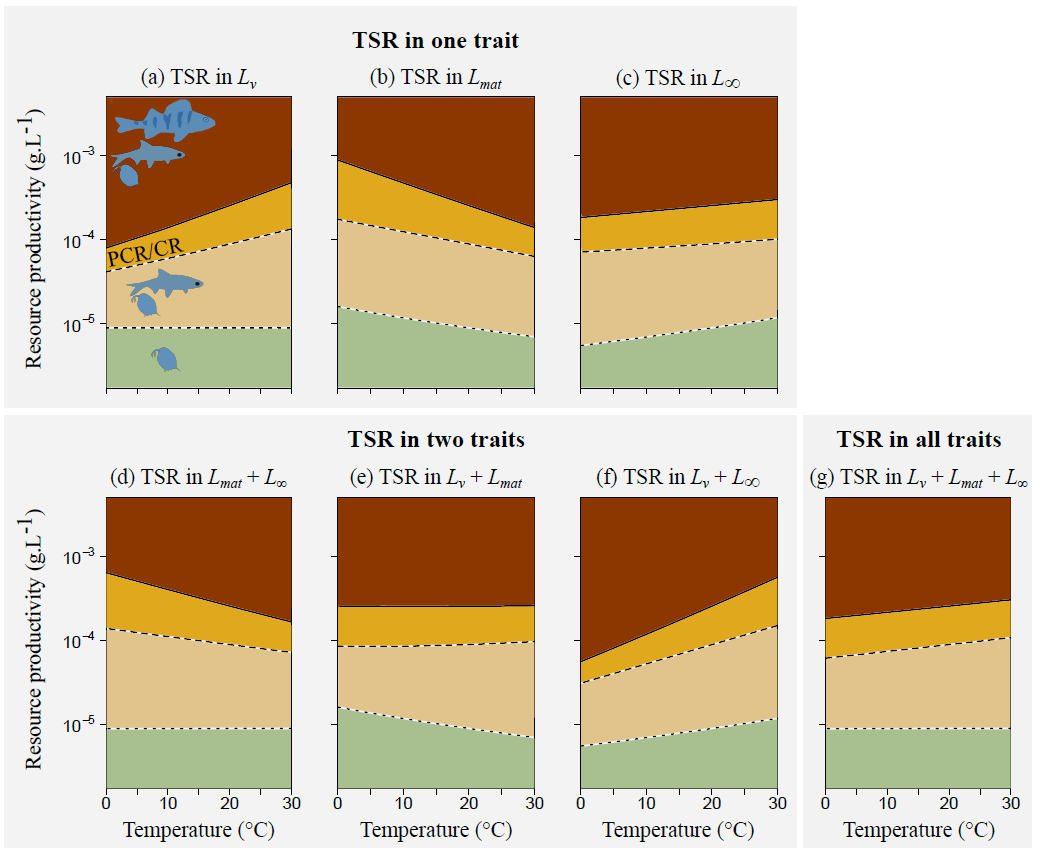
**Fig. S4. Influence of TSR on community structure along gradients of temperature and habitat productivity**. TSR affects one (a-c), two (d-f) or all three (g) measures of body size. Colours, line types and community structure as in Fig. 2.

**Fig. S5. Influence of TSR and temperature-dependent vital rates on community structure along gradients of temperature and habitat productivity.** The panels illustrate the 3x3 combinations of TPCs and TSR in consumer traits, predator traits and in consumer and predator traits together. TPCs implemented in (a-c) consumer only, (d-f) predator only, (g-i) consumer and predator; TSR implemented in (a, d, g) consumer only, (b, e, h) predator only, (c, f, i) consumer and predator. Colours, line types and community structure as in Fig. 2. Scenarios in panels (a), (e) and (i) corresponds to Scenarios 8-10 in Fig. 2.


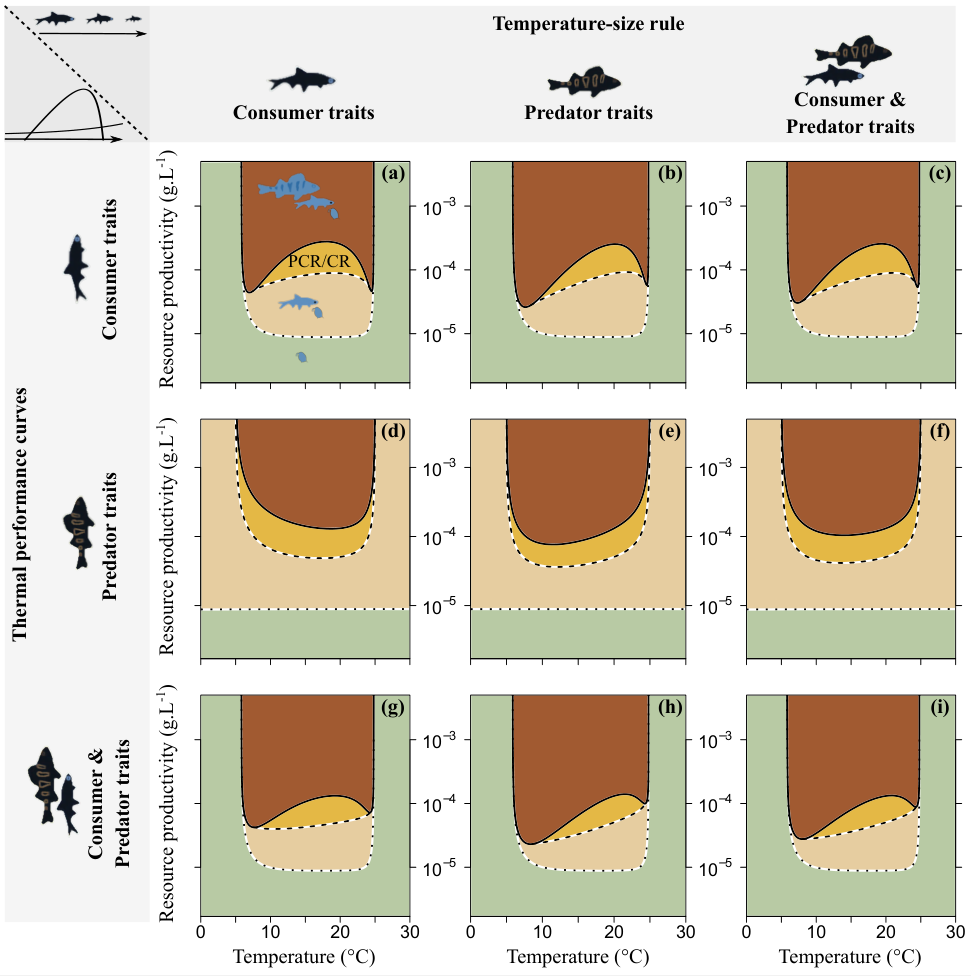


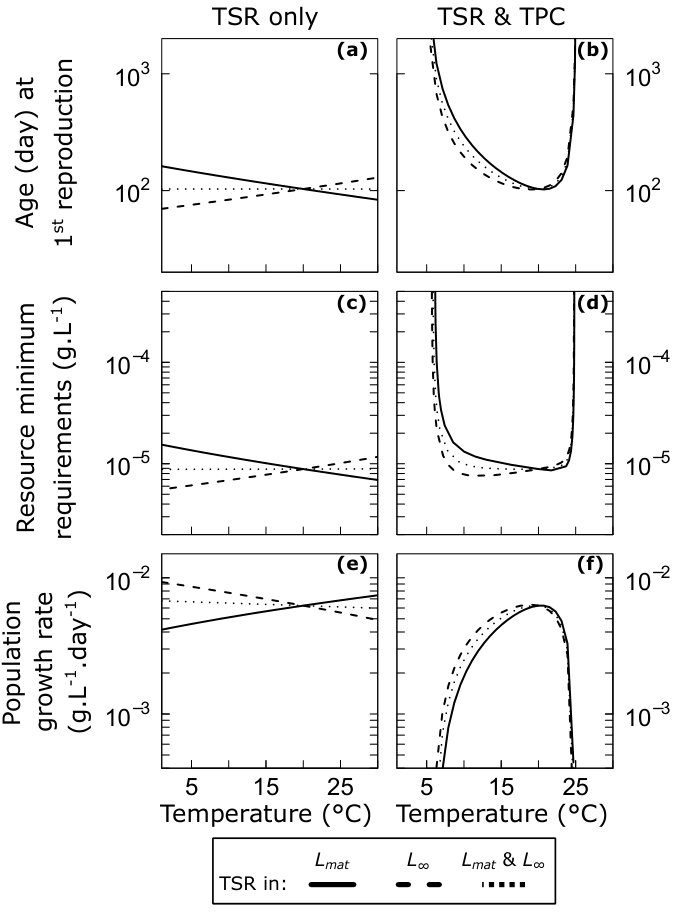
**Fig. S6. Effects of warming on consumer life history, minimum resource requirements and population growth rate.** (a-b) Age at first reproduction, (c-d) minimum resource requirement of consumers *K_C_* and (e-f) population growth rate. The effects involve (a, c, e) only TSR in consumer maturation size (*l_mat_*, solid lines), asymptotic size (*l_∞_*, dashed lines), and both sizes (dotted lines) or (b, d, f) TSR combined with temperature-dependent vital rates (TPC). Age at first reproduction and population growth rate calculated at a fixed, intermediate habitat productivity *K* = 10^-4^ g.L^-1^_._


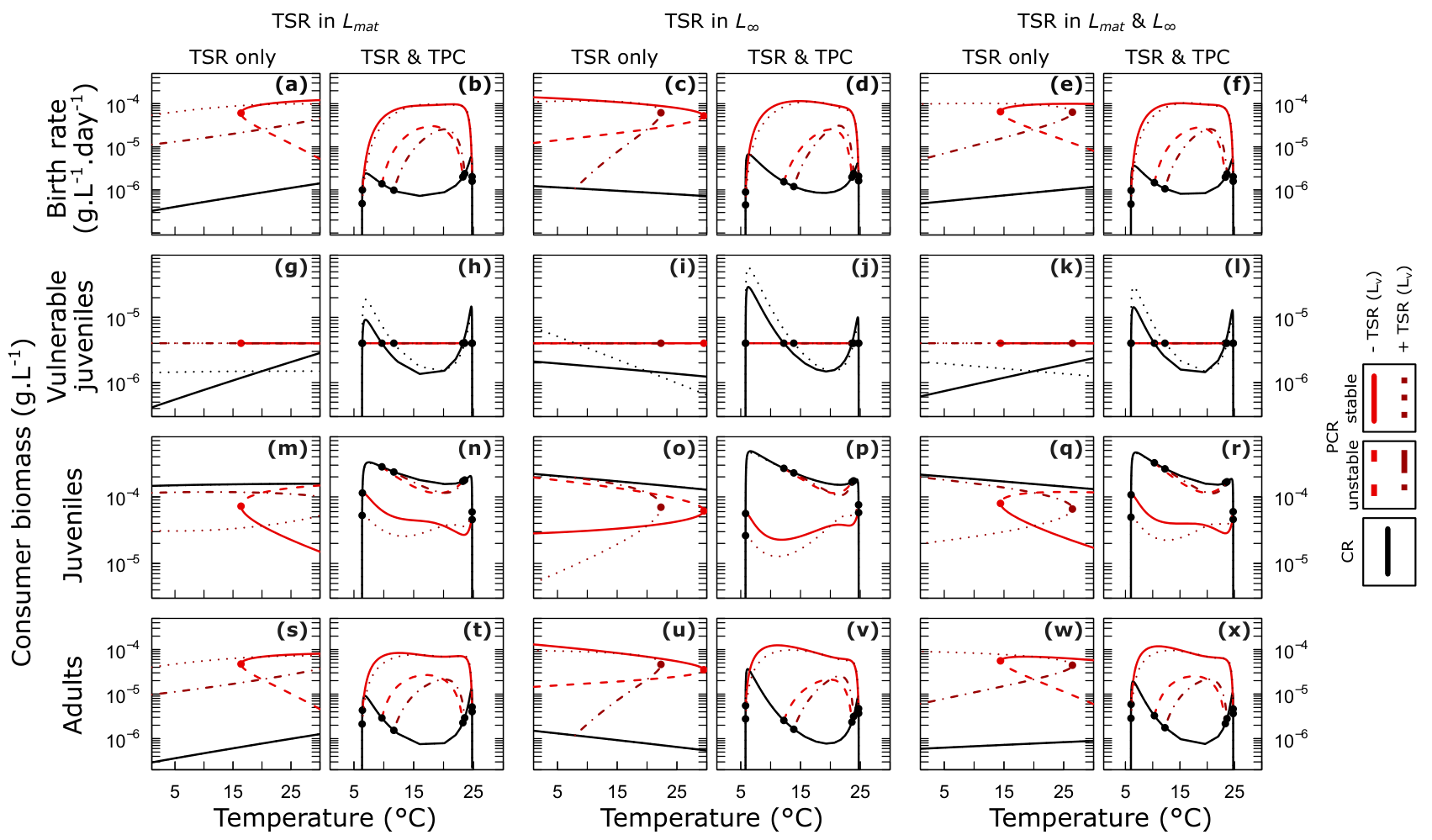
**Fig. S7. Temperature dependence of consumer birth rate and stage-specific biomasses** for a fixed habitat productivity at intermediate level, *K* = 10^-4^ g.L^-1^. Consumer per-capita birth rate (a-f) and the biomass of (g-l) vulnerable juveniles exposed to predation, (m-r) invulnerable juveniles and (s-x) adults. The scenarios involve TSR in *l_mat_* (columns 1 and 2), *l_∞_* (columns 3 and 4) and both *l_mat_* and *l_∞_* (columns 5 and 6). Columns 1, 3 and 5 illustrate the indirect effect of temperature through TSR; columns 2, 4 and 6 illustrate the combined effects of temperature (TSR & TPC). Community structure includes consumer-resource (‘CR’, black lines) and the tri-trophic chain (‘PCR’, red lines). Line types for the tri-trophic chain: solid line = stable equilibrium with temperature-independent *l_v_*, dotted line = stable equilibrium with temperature-dependent *l_v_*; dashed line = unstable equilibrium with temperature-independent *l_v_*; dot-dashed line = unstable equilibrium with temperature-dependent *l_v_*. Black points = invasion of top predator, red points = collapse of top predator. Corresponding community transitions: Fig. S4ae (column 1), Fig. S4cf (column 3), Fig. S4dg (column 5), Fig. 2gi (column 6). See Text S3 for details.

**Fig. S8. Cumulative biomass proportions of consumer stages across temperature gradient** for a fixed habitat productivity at intermediate level, *K* = 10^-4^ g.L^-1^ as in Figs S6 and S7. Stage-specific biomass proportions (%) in a stable consumer-resource equilibrium (a-l) and in a stable predator-consumer-resource equilibrium (P-C-R) (m-x) without TSR in predator that does not affect *L_v_* (a-f, m-r) and with TSR in predator that affects *L_v_* (g-l, s-x) are involved. Colours: light grey = juveniles vulnerable to predation, dark grey = non-vulnerable juveniles, white = adults. Black line at 100% added to improve readability. Vertical dotted lines = limits to predator persistence.


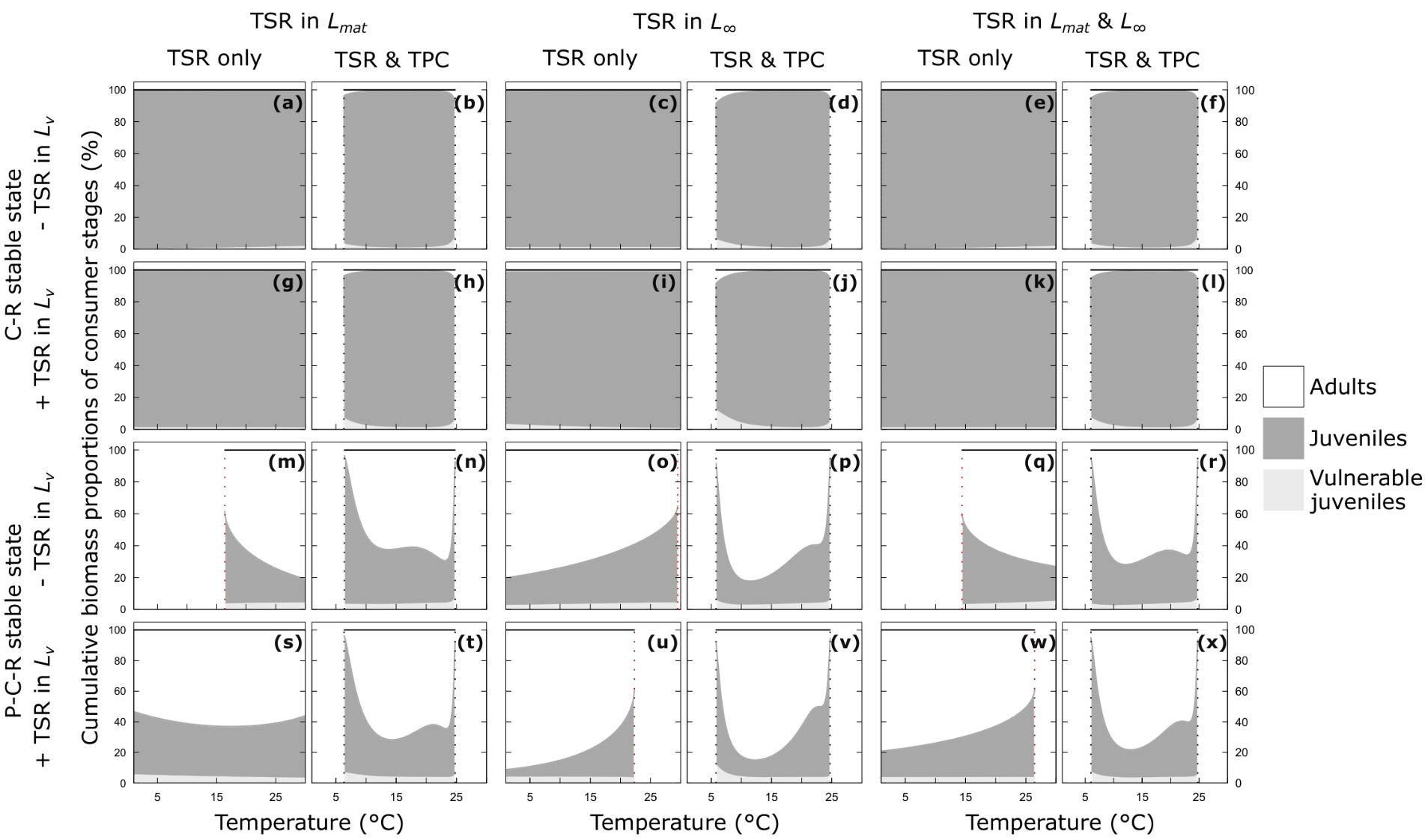

Supplement: Supplementary file 1 — Data S1. [file ELE-27-0-s001.docx]
